# Baculovirus Superinfection: A Probable Restriction Factor on the Surface Display of Proteins for Library Screening

**DOI:** 10.1371/journal.pone.0054631

**Published:** 2013-01-24

**Authors:** Xiaodong Xu, Yuanrong Chen, Yu Zhao, Xiaofen Liu, Beitao Dong, Ian M. Jones, Hongying Chen

**Affiliations:** 1 College of Life Sciences, Northwest A&F University, Yangling, Shaanxi, P. R. China; 2 School of Biological Sciences, University of Reading, Reading, United Kingdom; Wuhan Bioengineering Institute, China

## Abstract

In addition to the expression of recombinant proteins, baculoviruses have been developed as a platform for the display of complex eukaryotic proteins on the surface of virus particles or infected insect cells. Surface display has been used extensively for antigen presentation and targeted gene delivery but is also a candidate for the display of protein libraries for molecular screening. However, although baculovirus gene libraries can be efficiently expressed and displayed on the surface of insect cells, target gene selection is inefficient probably due to super-infection which gives rise to cells expressing more than one protein. In this report baculovirus superinfection of *Sf*9 cells has been investigated by the use of two recombinant multiple nucleopolyhedrovirus carrying green or red fluorescent proteins under the control of both early and late promoters (vAcBacGFP and vAcBacDsRed). The reporter gene expression was detected 8 hours after the infection of vAcBacGFP and cells in early and late phases of infection could be distinguished by the fluorescence intensity of the expressed protein. Simultaneous infection with vAcBacGFP and vAcBacDsRed viruses each at 0.5 MOI resulted in 80% of infected cells co-expressing the two fluorescent proteins at 48 hours post infection (hpi), and subsequent infection with the two viruses resulted in similar co-infection rate. Most *Sf*9 cells were re-infectable within the first several hours post infection, but the re-infection rate then decreased to a very low level by 16 hpi. Our data demonstrate that *Sf*9 cells were easily super-infectable during baculovirus infection, and super-infection could occur simultaneously at the time of the primary infection or subsequently during secondary infection by progeny viruses. The efficiency of super-infection may explain the difficulties of baculovirus display library screening but would benefit the production of complex proteins requiring co-expression of multiple polypeptides.

## Introduction

The baculovirus expression vector system (BEVS) is one of the most powerful and widely used platforms for the production of heterologous proteins in insect cells. Baculoviruses have a large circular double-stranded DNA genome that permits large DNA insertions and can be easily propagated and grown to high titers [1], [2]. Infection of insect cells with recombinant baculoviruses containing foreign genes under the control of strong baculovirus promoters (polyhedron or p10) has become a rapid and robust method for target protein production [1], [2]. As insect cells perform extensive post-translational modifications, such as glycosylation [3], phosphorylation [4] and disulfide bond formation [5] among others, BEVS is especially valuable for the expression of many complex eukaryotic proteins whose proper folding and biological activity requires post-translational modifications.

More recently, efforts have been made to develop BEVS as a eukaryotic platform for the display of complex proteins on the surface of budded virons, or insect and mammalian cells, for the purposes of antigen presentation, gene delivery or molecular screening [2], [6]. To date, baculoviruses have been successfully engineered for the surface display of numerous proteins and this technology has been used extensively in the production of vaccine immunogens [7] and in targeted gene delivery [8]. However, relatively few reports on the use of baculovirus display system for library screening have been published [9]–[12] despite the fact that the high expression levels and high titers possible suggest baculoviruses as a powerful tool for library generation and selection [2], [6].

We have reported the construction of a maize cDNA library [12] using baculovirus *Autographa californica* multiple nucleopolyhedrovirus (*Ac*MNPV) display system. Pools of proteins were successfully expressed and displayed on the surface of infected insect cells and cells expressing target proteins could be selected using magnetic or fluorescent activated cell sorting (FACS) technologies. However, enriching cells expressing individual proteins has proven more difficult than expected and viruses containing target genes were difficult to separate from other library members even after several rounds of selection. Insect cells are able to be infected by multiple baculoviruses and co-infection of insect cells with several recombinant viruses has been exploited in structure-function studies for the production of higher order proteins and protein complexes [13]. Therefore, we speculated that the failure to select cells carrying individual markers from expression libraries could be the result of a high level of super-infection with multiple viruses and that such super-infectivity might impede the development of efficient library screening.

To examine this possibility, we report here the engineering of two recombinant *Ac*MNPVs expressing green and red fluorescent proteins, and their use to investigate baculovirus super-infection of *Spodoptera frugiperda* (*Sf*9) cells. The aim of the study was to understand if super-infection is common and if it could act as a constraint on library screening.

## Materials and Methods

### Cells and Viruses

*Sf*9 cells (Invitrogen) were maintained at 27°C in SFX-INSECT medium (Thermo Scientific HyClone) with 2% fetal bovine serum (Thermo Scientific HyClone). *Ac*MNPV Bacmid was used in this study as modified by Zhao *et al*. [14].

### Construction of Recombinant Baculoviruses

The gene encoding enhanced green fluorescent protein (EGFP) was amplified by PCR from plasmid pCDNA3.1-EGFP (Invitrogen), using primers GFP-F (TAATCCATGGTGAGCAAGGGCGAG) and GFP-R (GCAACTCGAGCTTGTACAGCTCGTCC) and cloned into pBac™-5 (Novagen) between the *Nco*I and *Xho*I sites. Similarly, the *Discosoma* sp. Red (DsRed) gene was amplified from pDsRed-N1 (Clontech), using primers DsRed-F (TAATCGTCTCCCATGGCCTCCTCCGAGAAC) and DsRed-R (GCAACTCGAGCAGGAACAGGTGGTGG) and also cloned into pBac™-5 between the *Nco*I and *Xho*I sites. The resulting constructs were co-transfected with linearized Bacmid DNA into *Sf*9 cells using Fugene HD Transfection Reagent (Roche). Recombinant baculoviruses expressing green and red fluorescent proteins generated by homologous recombination were harvested at 4 days post infection (dpi) and labelled as vAcBacGFP and vAcBacDsRed.

### Baculovirus Titration and Genome Copy Quantification

Baculovirus titer was determined by end-point dilution assay in 96-well plates. Recombinant baculoviruses were serially diluted 10 fold in SFX medium to a final dilution of 10^−8^. Virus titer was calculated by monitoring and counting wells containing EGFP or DsRed foci at 2 dpi.

The DNA copy number of the viral stock was determined by quantitative PCR. Viral DNA was extracted using UNIQ-10 viral DNA extraction kit (Sangon Biotech) as described by the manufacturer’s protocol. PCR primers 1629FO (5′-AACTTGCCAAATCTTGTAGCAGC-3′) and 1629RO (5′-CGTGTTTACGTCGAGTCAATTGTAC-3′) were used to amplify the baculovirus 1629 gene and yield a 152 bp product. pTriEx1.1 (Novagen) plasmid DNA was serially diluted from 10^9^ to 10^3^ copies/ml and served as standards. Each PCR reaction contained 12.5 µl of SYBR *Premix Ex Taq* II (Takara). Quantitative PCR was performed in a BIO-RAD CFX96 thermocycler using cycling conditions of 94°C for 30 s followed 40 cycles of 95°C for 5 s, 60°C for 30 s. For each run, triplicates of standard DNA at known concentration, viral DNA samples and no template controls were simultaneously subjected to analysis. A standard curve generated by the standards was used to calculate the DNA copy numbers in the viral samples using the software “CFX_Manager”.

### Fluorescence Microscopy

Microscopy was carried out using an Olympus CKX41 microscope equipped with appropriate filters. DP2-BSW software was used for image acquisition and processing.

### Flow Cytometry

Infected *Sf*9 cells were collected by centrifugation for 2 min at 2,000 rpm, then washed and resuspended in phosphate-buffered saline (PBS). Data collection was performed on a CyFlow® Cube 6 flow cytometer (Partec) using CyView 8.5 software at an excitation wavelength of 488 nm. Cells expressing EGFP were detected in the FL1 channel at emission wavelength of 530 nm. Cells expressing DsRed were identified in the FL2 channel at emission wavelength of 585 nm. Data were collected from at least 4×10^4^ cells for each sample and data analysis was performed offline using FCS Express 4.

## Results

### Time Course for the Expression of Fluorescent Proteins

To explore the infection and superinfection potential of baculoviruses, recombinant *Ac*MNPVs (vAcBacGFP and vAcBacDsRed) expressing green and red fluorescent proteins respectively were constructed using the vector pBac™-5, which contains a modified gp64 tandem promoter including both an immediate early promoter for expression immediately after infection and a late promoter for continued expression in the late phase of infection. *Sf*9 cells were infected with each recombinant *Ac*MNPV at a multiplicity of infection (MOI) of 2 and fluorescent protein expression was observed using fluorescence microscopy at 8, 24, 48 and 72 hours post infection (hpi).

**Figure 1:**
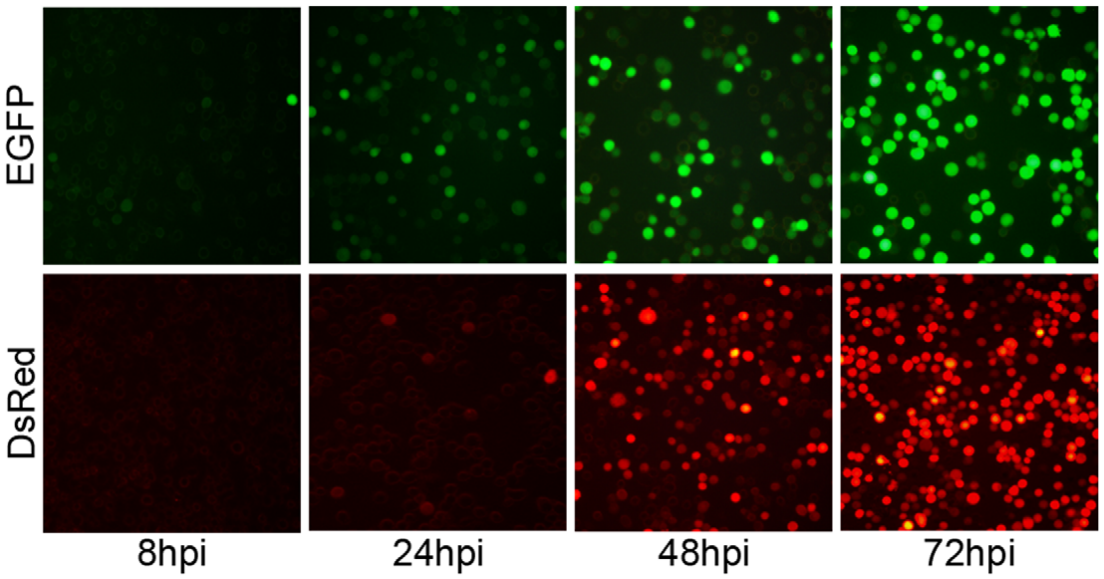
Time course for the expression of fluorescent proteins. *Sf*9 cells were infected with recombinant *Ac*MNPVs carrying fluorescent marker genes at 2 MOI. Images were taken at 8, 24, 48 and 72 hpi, using fluorescent microscope. EGFP: cells infected with vAcBacGFP; DsRed: cells infected with vAcBacDsRed.

EGFP was observed in cells infected with vAcBacGFP as early as 8 hpi and about half the population was green at 24 hpi, although the mean fluorescence intensity was still low. By 48 hpi, most cells were green and the fluorescence intensity continued to increase to 72 hpi (Fig. 1).

For cells infected with vAcBacDsRed, as DsRed is a tetramer and maturation of the fluorophore is slow [15], fluorescent cells were only visible at 24 hpi (Fig. 1). However, the population of red fluorescent cells rose to similar numbers as those expressing EGFP by 48 hpi when infected with the same MOI (Fig. 1). Thus vAcBacDsRed can be used together with vAcBacGFP for detection of late phase infection and co-infection in further studies.

### Multiplicity of Infection

**Figure 2:**
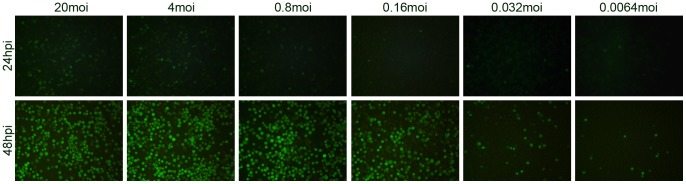
Microscopic analysis of the infection efficiency with vAcBacGFP at different multiplicities of infection. *Sf*9 cells were infected with 5 times serially diluted vAcBacGFP viruses, starting from an MOI of 20. The infection was observed and recorded using fluorescent microscope at 24 and 48 hpi.

As cells infected with vAcBacGFP could be detected as early as 8 hpi, *Sf*9 cells were infected with fivefold serially diluted viruses starting at an MOI of 20 to investigate the efficiency of baculovirus infection. Expression was observed and recorded using fluorescence microscopy at 24 and 48 hpi as before (Fig. 2). As expected, the fluorescence intensity was much greater at 48 hpi compared with the images at 24 hpi, even in the cases of low MOI (0.032 and 0.0064) at 48 hpi compared to high MOI (20 and 4) at 24 hpi. Thus, the combination of the initiation of the stronger late promoter and prolonged accumulation of fluorescent proteins products from the start of infection contributes to the high fluorescence intensity of the cells in late phase of infection.

**Figure 3:**
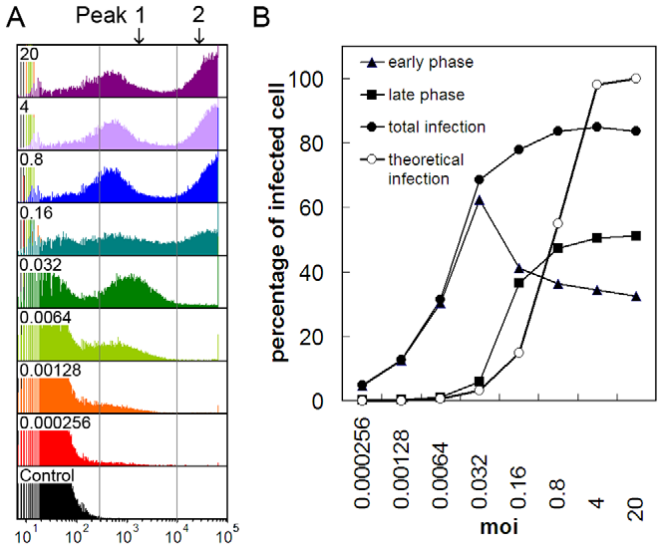
Flow cytometry analysis of cells infected with vAcBacGFP at different multiplicities of infection at 48 hpi. *Sf*9 cells were infected with 5 times serially diluted vAcBacGFP viruses, starting from an MOI of 20. A. Fluorescence histograms. From top to bottom: cells infected at 20, 4, 0.8, 0.16, 0.032, 0.0064, 0.00128, 0.000256 MOI, and uninfected cells as control. B. Comparison of the infection efficiency at different multiplicities of infection. Curves were drawn from the flow cytometry data. Early phase: fraction of cell population in peak 1; late phase: fraction of cell population in peak 2; total infection: fraction of cell population in peak 1 plus peak 2; theoretical infection: theoretical infection level based on the Poisson distribution.

Flow cytometry of the infected cells at 48 hpi clearly showed that the population of infected cells could be separated into two groups (Fig. 3A). A high fluorescence group (peak 2) reflecting cells in the late phase of infection and a low fluorescence group (peak 1) representing cells in the early phase of infection due to secondary infection. The data from the flow cytometry analysis are summarized in Figure 3B.

The percentage of infected cells in the cultures above 0.032 MOI were all greater than 60%, indicating that many virions had been released from primary infected cells, and both the primary and the secondary infection contributed to the high percentage of overall infection at 48 hpi (Fig. 3B). In low MOI infections (MOI = 0.032) only 6.01% of cells were in the late phase of infection by 48 hpi with 62.48% cells in the early phase of infection, a total infection rate of 68.49%. For cells infected at MOI>0.16 the ratio of cells in the late phase of infection increased slightly with higher multiplicity. However the cell population in the early phase decreased accordingly showing that adding viruses to a MOI>0.16 did not result in better infection overall. On the contrary, the number of infected cells in the culture at 20 MOI were actually slightly less than at 4 MOI consistent with the observations of Wu *et al*., who showed that cell viability decreased rapidly as the MOI increased in the range of 0.5–10 [16]. Using quantitative PCR, we checked the DNA copy number in the virus stocks, and found that 1 MOI represented 30–40 viral DNA copies for both vAcBacGFP and vAcBacDsRed, implying the existence of defective viruses in the inoculum. The entry of too many virus particles may lead to inhibition of virus replication or collapse of the infected cell, thus resulted in lower production of protein expression as well as progeny viruses for secondary infection.

For the cultures below 0.16 MOI, the populations of cells in late phase of infection (peak 2) were all higher than the theoretical value based on the Poisson distribution (Fig. 3B) demonstrating that secondary infection starts very early. On the contrary, at MOIs of 4 and 20, only 50.60% and 51.12% of cells respectively were in late phase of infection (Fig. 3B), although in theory most cells should have been infected by at least one virus particle at time zero. It is possible that a subset of the cell population is more susceptible to infection and/or that the presence of defective viruses or the inhibition of virus replication due to high load of virus could have lowered the infection rate.

### Co-infection with vAcBacGFP and vAcBacDsRed

**Figure 4:**
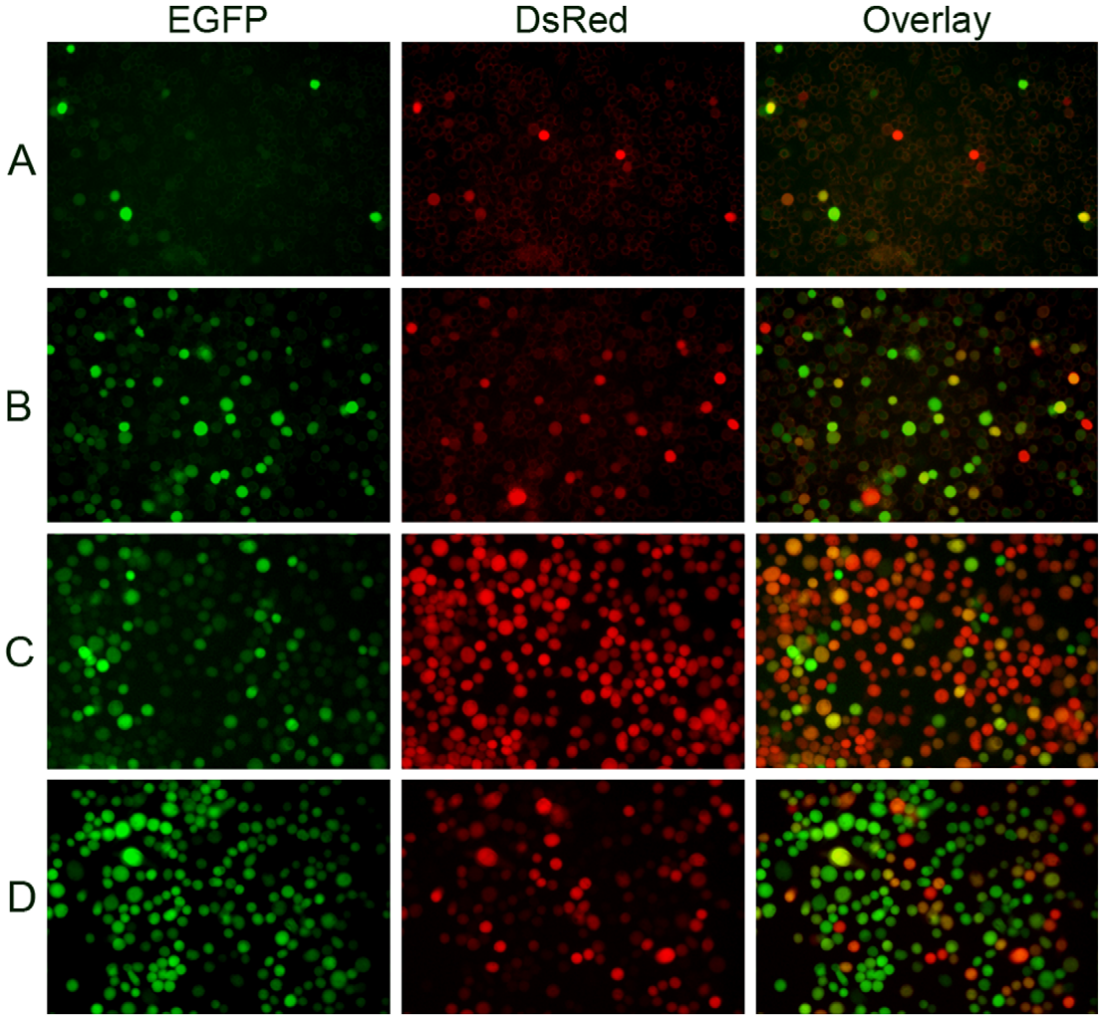
Microscopic analysis of *Sf*9 cells co-infected with vAcBacGFP and vAcBacDsRed. *Sf*9 cells were simultaneously infected with vAcBacGFP and vAcBacDsRed viruses. Protein expression was observed 48 hpi. Many cells were found co-expressing red and green fluorescent proteins even at low multiplicity. A. Cells were infected with 0.05 MOI of each virus. B. Cells were infected with 0.5 MOI of each virus. C. Cells were infected with vAcBacGFP and vAcBacDsRed at a ratio of 1∶4 respectively. D. Cells were infected with vAcBacGFP and vAcBacDsRed at the ratio of 4∶1 respectively.

To study the super-infection of baculoviruses by fluorescence microscopy, *Sf*9 cells were simultaneously infected with equivalent MOIs of vAcBacGFP and vAcBacDsRed viruses and protein expression was observed 48 hpi. Two different MOI values were used, 0.5 and 0.05, and under both circumstances, cells co-expressing red and green fluorescent proteins were observed among the infected cells (Fig. 4 A and B). This result clearly showed that these cells were super-infectable even when the cells were infected with baculoviruses at very low multiplicity.

**Figure 5:**
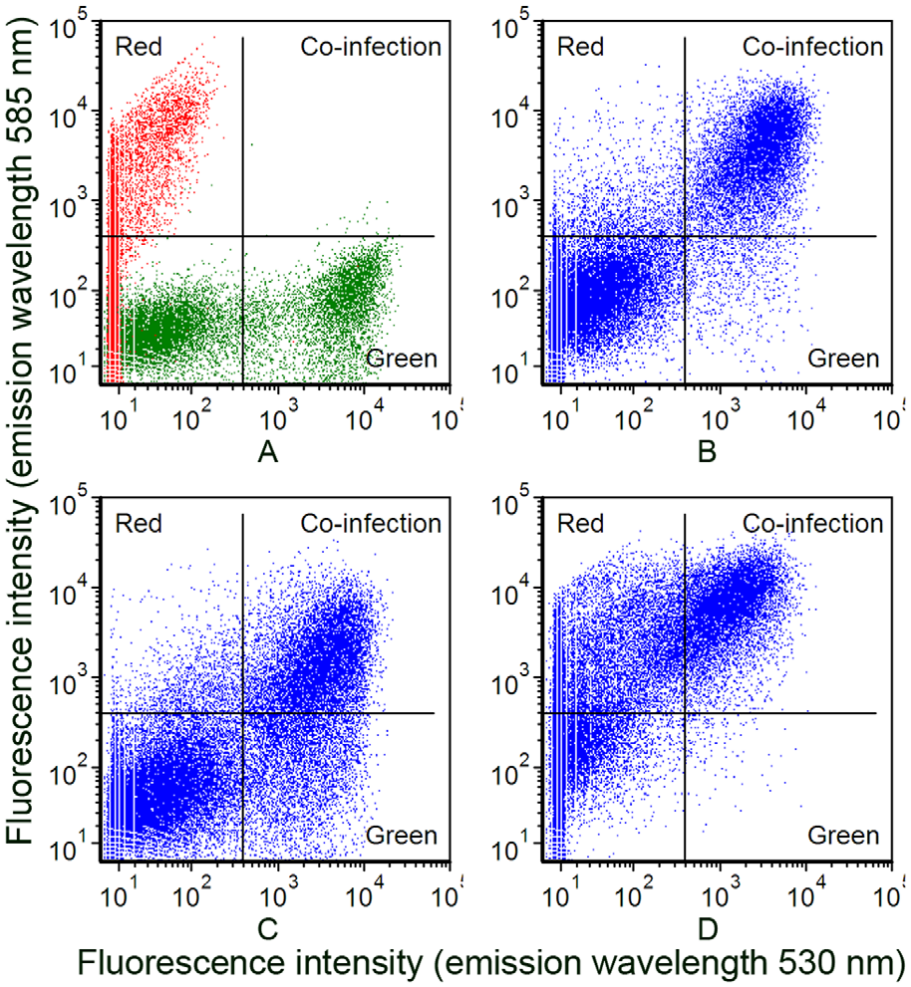
Detection of cell populations co-expressing EGFP and DsRed by flow cytometry. A. Individually infected cells. *Sf*9 cells individually infected with 1 MOI of vAcBacGFP and vAcBacDsRed were mixed at 48 hpi and used as the reference to gate the two-colored and single-colored cell subsets. B. Cells co-infected with vAcBacGFP and vAcBacDsRed at the MOI of 0.5 each. C. Cells co-infected with 0.8 MOI of vAcBacGFP and 0.2 MOI of vAcBacDsRed. D. Cells co-infected with 0.2 MOI of vAcBacGFP and 0.8 MOI of vAcBacDsRed.

To investigate co-infection further cells were infected with vAcBacGFP and vAcBacDsRed at different ratios and the outcome of infection was assessed by flow cytometry (Fig. 5). In all cases, the total amount of virus used for the infections was maintained at an MOI = 1. Cells separately infected with vAcBacGFP or vAcBacDsRed were mixed and used as the reference to gate the two-colored and single-colored cell subsets by flow cytometry analysis (Fig. 5A). When the viruses were used at a 1∶1 ration in infection (Fig. 5B), ∼25% cells were expressing at 48 hpi, with cells co-expressing green and red fluorescent proteins accounting for ∼20% of the total, suggesting that most infected cells tend to take up more than one virus at the time of infection. When the ratio of the two viruses were adjusted to 1∶4 or 4∶1 green to red respectively, the intensity of red and green fluorescence shifted accordingly with the varied proportion of input viruses but still about half of the infected cells were two-colored (Fig. 5 C and D). In this experiment, to avoid miscounting the strongly single-colored cells as dually infected, the cut-off for the fluorescence overlap was set quite high so that only strongly fluorescent cells were counted as positive. Therefore, the population of two-colored cells was likely underestimated as co-expressing two fluorescent proteins in co-infected cells may result in delayed accumulation or less yield of each protein.

The fluorescence images confirmed that the majority of two-colored cells infected with higher ratio of vAcBacDsRed were closer to red, while the two-colored cells infected with more vAcBacGFP viruses were closer to green. The expression level of each fluorescent protein in an individual cell was correlated with the number of each virus taken up by the cell. This result also suggested that each co-infected cell had taken up a number of viruses.

**Figure 6:**
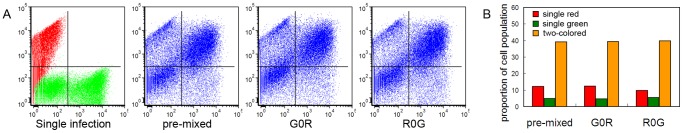
Comparison of cell infection using pre-mixed vAcBacGFP and vAcBacDsRed with infections using sequentially added viruses. A. Flow cytometry data for protein expression at 2 days post infection. Single infection: *Sf*9 cells were infected individually with vAcBacGFP and vAcBacDsRed then mixed and used as the reference to gate the two-colored and single-colored cell subsets; Pre-mixed: vAcBacGFP and vAcBacDsRed viruses were mixed at a ration of 1∶1, stored at 4°C for 1 day and then used to infect *Sf*9 cells; G0R: 1 MOI of vAcBacDsRed was applied to *Sf*9 cell monolayer, mixed well and held for 1 min, before adding 1 MOI of vAcBacGFP; R0G: 1 MOI of vAcBacGFP was applied to *Sf*9 cell monolayer, mixed well and held for 1 min, before adding 1 MOI of vAcBacDsRed. B. Comparison of the fluorescent cell populations. Bars reflect the flow cytometry data shown in A.

As the co-infection experiments used pre-mixed vAcBacGFP and vAcBacDsRed and the two viruses could have aggregated with each other before entering a same host cell, we also investigated the population of single infected and co-infected cells using sequentially added viruses as well as pre-mixed virus stocks. The simultaneous infection did not result in higher co-infection rate than the two successive infections (Fig. 6) suggesting that multiple virus entry into the same cell was not due to virus clumping in the inoculum.

### Re-infection of *Sf*9 Cells

**Figure 7:**
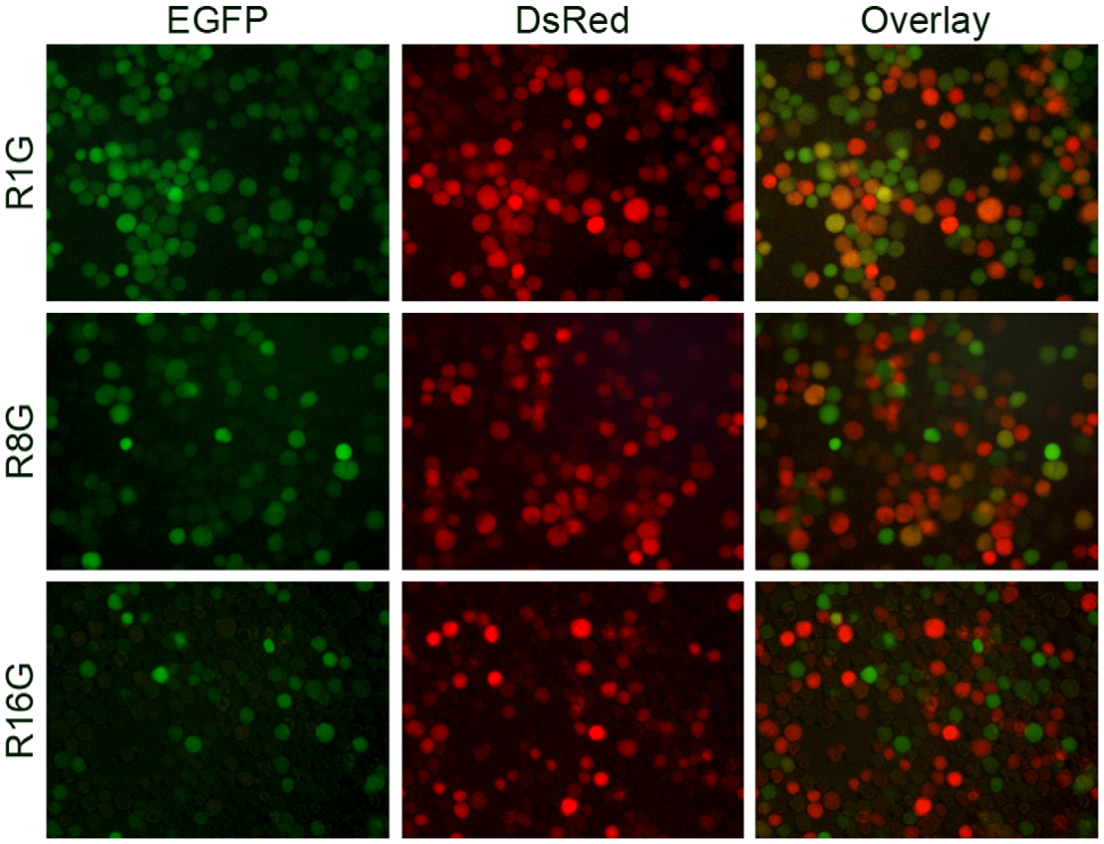
Re-infection of *Sf*9 cells with homologous baculoviruses. *Sf*9 cells were primarily infected with 1 MOI of vAcBacDsRed, and subsequently infected by 1 MOI of vAcBacGFP at an interval of 1 (top panel), 8 (middle panel) or 16 (bottom panel) hours. Protein expression was monitored 48 hpi of vAcBacDsRed by fluorescent microscopy.

As the superinfection of *Sf*9 cells late in infection implies that secondary produced virus can superinfect already infected cells, previously infected cells were tested for re-infection. *Sf*9 cells were initially infected with vAcBacDsRed at an MOI of 1 and subsequently infected by vAcBacGFP, also at an MOI of 1, at intervals of 1, 8 or 16 hours. Protein expression was monitored at 48 hours post primary infection by fluorescence microscopy. When *Sf*9 cells were super-infected at 1 or 8 hours post initial infection, a number of cells co-expressing green and red florescent proteins were observed (Fig. 7), indicated that these cells were readily super-infectable. In contrast, very few two-colored cells were found when vAcBacGFP was added 16 hours after the primary infection, suggesting that *Sf*9 cells could no longer be re-infected or the re-infection was unproductive at 16 hpi.

**Figure 8:**
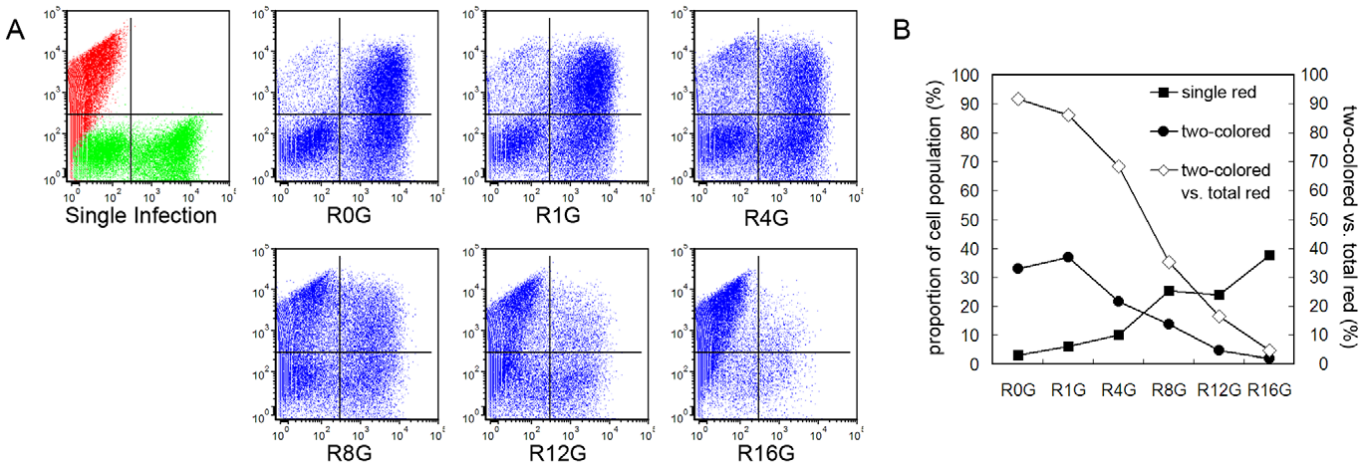
Flow cytometry analysis of *Sf*9 cells re-infected with homologous baculoviruses. *Sf*9 cells were primarily infected with 2 MOI of vAcBacDsRed, and subsequently infected by 4 MOI of vAcBacGFP at an interval of 0 (R0G), 1 (R1G), 4 (R4G), 8 (R8G), 12 (R12G) or 16 (R16G) hours. A. Protein expression was analyzed at 48 hpi of infection by vAcBacDsRed. Single infection: *Sf*9 cells were infected individually with vAcBacGFP and vAcBacDsRed then mixed at 48 hpi and used as the reference to gate the two-colored and single-colored cell subsets. B. Comparison of the fluorescent cell populations at 2 days post the first virus infection. Curves were drawn from the flow cytometry data shown in A. C. Comparison of the fluorescent cell populations at 3 days post the first virus infection.

To examine re-infectability of *Sf*9 cells in more detail, cells re-infected with 4 MOI vAcBacGFP at 0, 1, 4, 8, 12 or 16 hours after the primary infection of 2 MOI vAcBacDsRed were analyzed. The inocula were higher in this experiment to ensure a higher initial infection and sufficient opportunity for the second added virus to infect. When the viruses were added together, the single red cell number was very low (∼5% of infected cells) and greater than 90% of cells producing red fluorescent protein co-expressed green fluorescent protein (Fig. 8). As the time interval between primary and secondary virus addition increased, more single red cells and less single green and two-colored cells were observed. In the cells re-infected with vAcBacGFP at 16 hours post infection by vAcBacDsRed, most infected cells (∼89%) were singly red with only ∼4% infected cells co-expressing both fluorescent proteins, confirming that by 16 hpi *Sf*9 cells were generally not re-infectable.

## Discussion

In addition to direct protein expression, baculoviruses have been developed for the surface display of eukaryotic proteins in a similar manner to phage display for the selection of useful molecules from complex libraries. For example, a clone with increased binding ability to 2F5, a broadly neutralizing monoclonal antibody against HIV-1 gp41, was isolated from a library of 8000 variants displaying the 2F5 epitope with different surrounding amino acids [9]. Further, libraries of peptides bound to major histocompatibility complex (MHC) class I or class II were displayed on the surface of baculovirus infected insect cells and peptide antigen mimotopes selected from libraries using fluorescent multimeric soluble T-cell receptors (TCRs) [10], [11], [17]. Despite these examples however, further development of baculovirus displayed libraries appears restricted. Previous attempts to select baculoviruses expressing calreticulin or auxin binding protein from a maize cDNA library showed that while baculovirus infected cells expressing the target proteins could be enriched by magnetic sorting [12], the viruses recovered had not enriched the desired virus population. These findings indicate that *Sf*9 cells might be easily super-infectable and thus impede subsequent library screening.

Since it was engineered as a protein expression system in 1983 [18], BEVS has been very widely used for single recombinant protein production and for the study of protein–protein interactions and the co-infection of insect cells with multiple baculoviruses has been reported [13]. Nevertheless, little work has been done to investigate what happens within cells or cell culture during co-infection.

In this report, two recombinant *Ac*MNPVs carrying red or green fluorescent marker genes were used for the study of infection, co-infection and re-infection of *Sf*9 cells. Previously, Lee *et al*. used EGFP and DsRed to investigate baculovirus superinfection of *Drosophila* S2 cells [19] and GFP tagged VP2 and immunostained VP6 were monitored to optimize the simultaneous expression of rotavirus proteins for the production of rotavirus-like particles by baculovirus co-infection [20]. These studies used constructs driven solely by late promoters and expression was only detected later than 24 hpi. In the present study, the fluorescent genes were under the control of both early and late promoters and expression of EGFP by vAcBacGFP could be observed as early as 8 hpi (Fig. 1). Notably, as the late promoter is much stronger than the early promoter, vAcBacGFP infected *Sf*9 cells could be distinguished in the early and late phases of infection by two peaks in the flow cytometry analysis (Fig. 3A).

Flow cytometry analysis of vAcBacGFP infected cells at 48 hpi showed that the total infection percentage in the cultures infected with viruses at 0.032 MOI and above were all higher than 60%, and both the primary and the secondary infection contributed to the high percentage of infection. This result is consistent with the previous data obtained by Mena *et al*., where between 65 to 90% of the cells were expressing recombinant protein at 48 hpi, regardless of the MOI in a range from 0.1–20 [20]. For the cultures below 0.16 MOI, the populations of cells in the late phase of infection were all higher than the theoretical value based on the number of viruses added. Baculoviruses bud from infected cells as early as 10−12 hpi [21], [22] and it seems likely that some cells in the late phase of infection at 48 hpi were infected by viruses released by primary infected cells.

We also show that infected cells can continue to be infected by additional viruses for a considerable time. It has been shown previously that virus absorption by infected cells can occur up to 24 hpi albeit at a reduced rate [23] and re-infection is capable of protein expression up to 12 hpi [24]. Our results show that the re-infectability of *Sf*9 cells decreases over 16 hpi, with the proportion of initially infected cells co-expressing the second fluorescent protein dropping from 91% at 0 hpi to 35% at 8 hpi, 16% at 12 hpi, and only 4% by 16 hpi (Fig. 8). Previous reports have shown that baculovirus binding to insect cells is non-saturable [25], [26] suggesting that insect cells have a large number of receptors for *Ac*MNPV (10^5^ to 10^7^ per cell) [27] or that *Ac*MNPV binds to the plasma membrane directly [28]. As a result, uptake is not considered to be limited by receptor availability even when the level of defective virus particles in the inoculum is taken into account. However, the cellular machinery for viral DNA replication and protein production could be saturated after a given time of infection, effectively preventing a productive cycle for viruses that enter the cell at later times [13], [29].

Simultaneously infected vAcBacGFP and vAcBacDsRed viruses revealed that ∼80% of infected cells were co-expressing red and green fluorescent proteins when infected at a 1∶1 ratio. In our studies, we found that the status of *Sf*9 cells and different batches of virus stock marginally affected the overall infection rates but that the ratio of co-infected to overall infected population of cells were always around 80% when the two viruses were mixed at a ratio of 1∶1 and used at an MOI of 0.5 to 2. When the ratio of the two viruses changed to 1∶4 or 4∶1 about half of the infected populations were also co-infected suggesting there may exist a proportion of cells in a population that are more infectable than others. As simultaneous infection did not result in a higher co-infection rate than successive infection (Fig. 6), and primary infected cells remain re-infectable for up to 16 hours (Fig. 7 and 8), we speculate that a subset of *Sf*9 cells are more susceptible to baculovirus infection than others, and that this population can actively take up multiple virions during the early phase of infection or continuously following secondary infection by progeny viruses. In terms of a complex library of recombinant viruses, each infected insect cell could have a mixture of viruses expressing different proteins effectively meaning that selection of a target protein will not be as easy as in the phage display system where super-infection immunity effectively prevents bacteria from continuous re-infections. The previous cases of successful library screening may be attributed to starting from relatively small libraries [9], infecting cells with viruses at low multiplicity and selecting with stringent conditions including multiple rounds of single cell sorting [11].

While super-infection may impede the application of BEVS to library display and screening, our data highlight its suitability for the production of complex proteins such as immunoglobulin and virus-like particles and for the study of protein-protein interactions which require efficient co-expression of multiple polypeptides.
